# Supplementary material for: Ganaxolone, an approved therapy for CDKL5-deficiency disorder, is an inhibitor of PTP1B
Source: J Biol Chem. 2026 May 20;302(7):113178. doi: 10.1016/j.jbc.2026.113178 (PMC13311170; doi:10.1016/j.jbc.2026.113178)
Supplement: Supporting Figures and Tables [file mmc1.pdf]

Ganaxolone, an approved therapy for CDKL5-Deficiency Disorder, is an inhibitor of PTP1B

Imanol Zubiete-Franco[1, #], Qingting Hu[1,2, #], Steven R. Alves[1], and Nicholas K Tonks[1, *]

[1]Cold Spring Harbor Laboratory, Cold Spring Harbor, New York 11724, USA
[2]Graduate Program in Genetics, Stony Brook University, Stony Brook, NY, USA.

Supporting Information

List of Contents:

1. Response of 293T cells to EGF and response of SH-SY5Y cells to insulin and a chemically distinct PTP1B inhibitor (CPT-157633) ______________________________S2
2. Table of sgRNAs used in this study____S3

Supplemental Figure 1

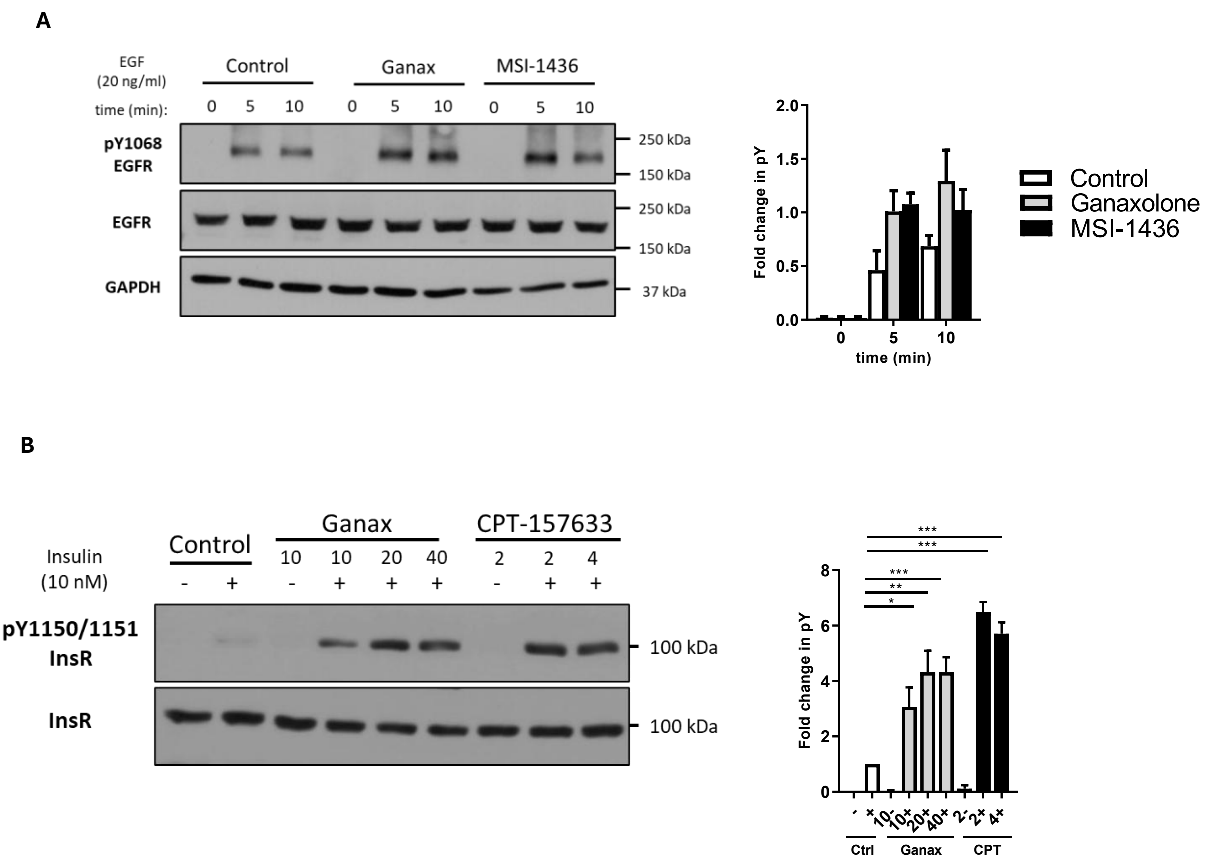

Supplemental Figure 1: A) Response of human 293T cells to EGF (20 ng/ml, 5/10 minutes) after 1 hour pre-treatment with vehicle (Control), ganaxolone (5 μM, Ganax), or MSI-1436 (4 μM, MSI-1436). **B)** Undifferentiated SH-SY5Y cells were pre-treated with drug (ganaxolone: 10-40 μM, CPT-157633: 2-4 μM) for 1 hour. Treated cells were then stimulated with 10 nM Insulin for 10 minutes, collected and probed for Insulin receptor phosphorylation. Immunoblot quantitation is shown on right. Ganax = ganaxolone, CPT = CPT-157633.

<u>Supplemental Materials and Methods</u>

<u>Supplemental Table 1</u>: List of sgRNAs used in the study.

GENE	SEQUENCE (5'-3')
PTPN1 (293T)	**A: CAGTGACTTCCCATGTAGAG**<br>**CTCTACATGGGAAGTCACTG**<br><br>**B: GACGTCTCTGTACCTATTT**<br>**AAATAGGTACAGAGACGT**
CDKL5 (SH-SY5Y)	**sg1: TACGAGAGCTTAAAATGCTT**<br>**AAGCATTTTAAGCTCTCGTA**<br><br>**sg2: AATGCTTCGGACTCTCAAGC**<br>**GCTTGAGAGTCCGAAGCATT**
PTPN1 (SH-SY5Y)	**GTCTTTCAGTTGACCATAGT**<br>**ACTATGGTCAACTGAAAGAC**

Sequences of the sgRNAs used in this study. The plasmid (pDG459) used to create PTP1B-KOs in 293Ts incorporates two sgRNAs (A and B) within the same plasmid. The two sgRNAs used in to create CDKL5-KO in SH-SY5Y were used to create two different clones.
